# 

**Published:** 1827-07

**Authors:** 


### THE MEDICO-CHIRURGICAL REVIEW.

*NEW SERIES.*

### VOLUME SEVEN.

[BEING VOL. XI. OF ANALYTICAL SERIES.]

CONDUCTED BY
ASSOCIATED PHYSICIANS AND SURGEONS;

AND SUPERINTENDED BY
JAMES JOHNSON, M.D.

MEMBER OF THE ROYAL COLLEGE OF PHYSICIANS OF LONDON, AND PHYSICIAN EXTRAORDINARY TO HIS ROYAL HIGHNESS THE DUKE OF CLARENCE.

Nec tibi quid liceat sed quid fecisse decebit
Occurrat mentemque domat respectus honesti.—CLAUD.

Surgeon Genl's Office LIBRARY. 57446 Washington, D.C.

LONDON:

*Printed by G. Hayden, Little College Street, Westminster.*

Published by S. Highley, 174, Fleet Street, and Webb Street, St. Thomas's Hospital; Baldwin, Cradock, & Joy, Pater-Noster Row; Kingsbury, Parbury, & Allen, Leadenhall Street; T. & G. Underwood, Fleet Street; Callow and Wilson, Princes St[illegible] Soho; Burgess & Hill, Windmill Street, Haymarket; Anderson, We[illegible]thfield; Cox & Son, and Jackson, Borough; J. Nimmo, Great Maze Po[illegible], Borough; Adam Black, Edinburgh; D. Lizars Princes Street, Edinburgh; Richard Griffin & Co., W. R. M'Phun, and David Allan & Co. Glasgow; Hodges & M'Arthur, Dublin; Messrs. Treuttel & Wurtz, Paris and Strasburg.

1827.

W1
ME 75T

### THE MEDICO-CHIRURGICAL REVIEW, AND JOURNAL OF PRACTICAL MEDICINE.

SECOND VOLUME,
[1st of JULY to 30th of SEPTEMBER,]
1827.

EDITED BY JAMES JOHNSON, M.D.
&c. &c. &c.

PUBLISHED BY S. HIGHLEY,
174, FLEET STREET,
AND WEBB STREET, ST. THOMAS'S HOSPITAL,
And all other Booksellers.

### BIBLIOGRAPHICAL RECORD;*

OR,

*Works received for Review between the 15th of March, and the 15th of June, 1827.*

1. A Treatise on Physiology applied to Pathology. By F. J. V. Broussais, M.D. &c. Translated from the French by John Bell, M.D. &c. and R. La Roche, M.D. &c. 8vo, pp. 559. Philadelphia, 1826.
☞ *An excellent work.*

2. Observations on the Causes and early Symptoms of Defects in the Form of the Spine, Chest, and Shoulders, and on the Means of correcting them: with Remarks on the different Methods pursued in this Country and on the Continent, in the Treatment of Distortions; being an enlarged Edition of the Papers lately published in the London Medical Journal. By John Shaw, Surgeon of the Middlesex Hospital; and formerly Lecturer on Anatomy. 8vo, pp. 132, plates vii. London. Longman's, 1827.

3. Thought not a Function of the Brain: a Reply to the Arguments for Materialism advanced by Mr. W. Lawrence, in his Lectures on Physiology. 8vo, pp. 80. Rivington, London, 1827.

4. A Treatise on the Diseases of Females. By Wm. P. Dewees, M.D. Adjunct Professor of Midwifery in the University of Pennsylvania, &c. 8vo, pp. 557. Philadelphia, 1826.

5. Clinical Observations on the Efficacy of Hydrochloruret of Lime, as a Remedy in certain Stages of Fever and Dysentery. By Robert Reid, M.D. Author of a Treatise on Tetanus and Hydrophobia, &c. 8vo, stitched, pp. 35. Hodges and M'Arthur, Dublin, 1827.

6. Dr. Hassel's Practical Observations on the General and Local Antiseptic Properties of the Chlorurets of the Oxides of Sodium and Calcium, in the Practice of Physic and Surgery; and for correcting the Effluvia of decomposed Animal Matter. 8vo, stitched, pp. 31. Price 2s. 6d.

7. Farming the Sick Poor. Observations on the Necessity of establishing a different System of affording Medical Relief to the Sick Poor: than by the Practice of contracting with Medical Men, or the Farming of Parishes. By J. F. Hulbent, M.R.C.S. &c. 8vo, stitched, pp. 51. Shrewsbury, 1827.

* We have to apologize to authors for any omissions or inaccuracies which may occur in the present Record. The paper on which we inscribe the works we receive was unfortunately mislaid at a late period of the quarter, and the list must of course be, in some degree, imperfect. If, however, gentlemen will send us the *titles* of those works which are unnoticed, they shall be inserted in our next. *Ed.*

8. Medical Botany; or, Illustrations and Descriptions of the Medicinal Plants of the London, Edinburgh, and Dublin Pharmacopœias, with those lately introduced into Medical Practice; comprising their Generic and Specific Characters; English, Provincial, and Foreign Appellations; a copious List of Synonymes; Botanical Descriptions; Natural History; Physical, Chemical, and Medical Properties and Uses: including also a Popular and Scientific Description of Poisonous Plants, particularly those that are indigenous to Great Britain and Ireland; with Figures coloured from Nature; the whole forming a complete System of Vegetable Toxicology and Materia Medica. By JOHN STEPHENSON, M.D. Graduate of the University of Edinburgh; and JAMES MORSS CHURCHILL, Esq. M.R.C.S. &c. Parts IV. and V. for April and May, 1827. John Churchill, London. 3s. 6d. each part.

---

9. A Treatise on Special and General Anatomy, in Two Volumes. By WILLIAM E. HORNER, M.D. Adj. Prof. Anat. Univ. Pennsylvania, &c. 8vo, bound, pp. 491—524. Philadelphia, 1826.

☞ *This seems to us a meritorious work. The author, in an amusing and clever preface, bears rather hardly upon Mr. John Bell. That gentleman, with all his faults, was no fool, and though he was somewhat given to hyperbole, and rather too fond of shewing up his brethren, we very much question whether his system of anatomy and physiology is not far preferable to many of the slip-slop productions of the present day. This censure does not apply to Dr. Horner, whose Treatise appears to us to be well and carefully written, and will, we are sure, prove highly useful to those who are studying their profession.*

---

10. Pathological and Practical Observations on Spinal Diseases: illustrated with Cases and Engravings. Also, an Enquiry into the Origin and Cure of Distorted Limbs. By EDWARD HARRISON, M.D. F.R.A.S. Ed. &c. pp. 294, plates xv. Underwoods', London, 1827.

☞ *In our next.*

---

11. A Treatise on Clinical Medicine, being a compendious and systematic Introduction to Practice, as contained in the Memoranda of J. R. BISCHOFF, M.D. Imperial Professor of Clinical Medicine, &c. From the German, by JOSEPH COPE, M.D. 12mo, pp. 280. London, Highley. 1827. Price 6s.

---

12. Laws of Physiology; translated from the Italian of Il Sig. Dott, B. Mojon, Professor Emeritus in the Royal University of Genoa, and Member of many learned Bodies. With additions, and a Physiological Table of Man. Dedicated by permission, to Sir Astley Paston Cooper, Bart. F.R.S. Surgeon to the King. By GEORGE R. SKENE, M.R.C.S. &c. 8vo, pp. lxxii.—125. Burgess and Hill, London, 1827.

☞ *The author of this translation is a very meritorious and well educated young surgeon. We hope he will hereafter appear in the character of author rather than translator, since the original matter in this volume is such as to raise hopes of higher labours from the same pen.*

---

13. Two Introductory Lectures on Materia Medica, delivered at the opening of the Course, and at the Commencement of the Vegetable Materia Medica, at Mr. Grainger's Theatre, Webb Street, Maze Pond, Borough, and at the New Medical School, Little Dean Street, Soho. By FRANCIS BOOTT, M.D. Octavo, pp. 70. Highley, London, 1827. Price 3s. 6d.

☞ *These Introductory Lectures disclose a mind highly gifted, and stored with the fruits of ample and well-directed researches.*

---

14. An Essay on Gout: in which its actual Predisponent, Proximate, and Exciting Causes are clearly defined; and its preventive and curative indications fully demonstrated, upon new Pathological Principles, which exhibit a more consistent, safe, and efficient Method of Treatment than any hitherto promulgated. To which are added, Observations on the Modus Operandi of Bath Waters in Gouty Habits. By P. P. P. MYDDELTON, M.D. &c. Fourth Edition. 8vo, pp. 97. Bath, 1827.

---

15. Elements of Physics, or Natural Philosophy, general and medical, explained independently of Technical Mathematics. By N. ARNOTT, M.D. of the

Royal College of Physicians. 8vo, pp. 611. Underwoods', London. 1827.

☞ *See our present number.*

16. The American Medical Recorder, Nos. 35 to 38, inclusive, July 1826 to April, 1827. *In exchange.*

☞ *We shall feel much obliged to Mr. Webster to include the Medical Recorder in any parcel which he may be sending to Mr. Millar, the spirited American bookseller, in London. We shall repay Mr. Millar his expenses. Sent separately by packets on board ship, the expense is considerable.*

17. The North American Medical and Surgical Journal, No. VI. *In exchange.* April, 1827.

18. The New York Medical and Physical Journal, quarterly, No. 21. April, 1827.

19. A Treatise on the Nature and Cure of Rheumatism; with Observations on Rheumatic Neuralgia, and on Spasmodic Neuralgia, or Tic Douloureux. By Charles Scudamore, M.D. F.R.S. Honorary Member of Trinity College, Dublin, &c. 8vo, pp. 589. London, 1827.

20. The Hunterian Oration, delivered before the Royal College of Surgeons in London, on Wednesday, Feb. 14, 1827. By H. Leigh Thomas, F.R.S. &c. 4to, pp. 28. 1827.

21. An Oration delivered before the Medical Society of London, on Thursday, March 8, 1827, (being the fifty-fourth anniversary) by Wm. Kingdon, Vice President, &c. 8vo, pp. 16. London, 1827.

22. An account of the apparatuses for the treatment of rheumatism and diseases of the skin, which have been constructed at the Dublin Skin Infirmary; illustrated by many Plates. By Wm. Wallace, M.R.I.A. Surgeon to the Charitable Infirmary of Dublin, and to the Infirmary for the Treatment of Rheumatism and Cutaneous Diseases in that City, &c. Second Edition. 4to, pp. 44. Dublin, 1827.

23. The Anatomy of Drunkenness. By Robert Macaish, Member of the Glasgow Medical Society. 8vo, pp. 56. M. R M'Phun, Glasgow, 1827.

☞ *If the Religious Tract Society were to distribute 30 or 40 thousand copies of the above pamphlet, they would probably do more good both to the bodies and souls of men in the lower orders of society, than by double the number of their most celebrated orthodox effusions.*

24. Some Account of the Science of Botany; being the substance of an Introductory Lecture to a Course on Botany, delivered in the Theatre of the Royal Institution of Great Britain. By John Frost, F.A.S. F.L.S. &c. (Dedicated by permission to the King.) 4to, pp. 17. London, 1827.

25. Commentaries on some of the more important of the Diseases of Females. In Three Parts. By Marshall Hall, M.D. F.R.S. E. &c. 8vo, pp. 376, plates viii. London, Longman's, 1827.

☞ *In our next.*

26. The New London Medical and Surgical Dictionary; including Anatomy, Chemistry, Botany, Materia Medica, Midwifery, Pharmacy, Physiology, &c. With the collateral Branches of Philosophy, Natural History, &c. By J. S. Forsyth, Surgeon, &c Author of the New London Medical Pocket-book, &c. 12mo, pp. 930. Sherwood, London, 1827. Price 15s. boards.

27. Journal des Progrès des Sciences et Institutions Médicales en Europe, en Amérique, &c. 2d volume, 1827. Paris, 1827. *In exchange.*

28. Some Observations on the Medicinal and Dietetic Properties of Green Tea, and particularly on the controlling influence it exerts over irritation of the Brain. By W. Newnham, Esq. Author of an Essay on Inversio Uteri, &c. pp. 32. stitched. London, 1827.

☞ *See Periscope.*

29. Reply to the "Additional Strictures" contained in the first number of the Quarterly Medical Review, &c. By Leonard Koecker. Pp. 35. London, 1827.

☞ *It was hardly worth Mr. Koecker's while to answer the defunct. The criti-*

*cism on Mr. Koecker's work was so clearly a venomous and jealous effusion, that it failed entirely in its object. Mr. K. has nothing to fear from it.*

30. A Clinical Lecture delivered to the Students of Surgery in the Royal Infirmary of Edinburgh, at the conclusion of the Winter Course for 1826-27. By George Ballingall, M. D.

☞ *One of Dr. Ballingall's cases, supposed colica pictonum, will be found noticed in our review of Andral this quarter. We shall probably notice some more of the cases in this able report in our next.*

31. The Life of Edward Jenner, M.D. L.L.D. F.R S. Physician Extraordinary to the King, &c. with Illustrations of his Doctrines, and Selections from his Correspondence. By John Baron, M.D. F.R.S. 8vo, pp. 608. London, Colburn, 1827. ☞ *In our present number.*

32. A Clinical Report of the Royal Dispensary for Diseases of the Ear. With Remarks on the Objects and Utility of the Institution. By John Harrison Curtis, Esq. Surgeon Aurist to His Majesty, &c. pp. 48. London, 1827.

33. A Case Book for registering Cases and Occurrences that may be considered important in Medical and Surgical Practice, with a Chart appended, as a Guide for taking Cases. Jackson, Borough, London.

☞ *This is the best form of case-book we have yet seen, and we strongly recommend it to every private practitioner who wishes to record, and profit by the records of his experience.*

34. Treatise on the Theory and Practice of Physic. By George Gregory, M.D. with Notes and Additions adapted to the Practice of the United States. By Nathaniel Potter, M.D. Professor of the Practice of Physic in the University of Maryland; and S. Colhoun, M.D. Member of the American Philosophical Society. In two volumes, 8vo, pp. 532—pp. 546. Philadelphia, 1826.

☞ *The notes and additions to this transatlantic republication of Dr. Gregory's Practice of Physic, do great credit to the talented editors, who have transplanted our able countryman's production to a foreign soil. They give us a good idea of the actual state of medical practice in America—and that idea is calculated to raise the character of the transatlantic profession wherever this edition may find its way.*

35. Observations upon the Origin and latent Period of Fever. By Henry Marsh, A.B. M.D. &c. Physician to Steeven's Hospital, &c. 8vo, pp. 80. (From the 4th vol. of the Dublin Hospital Reports.)

☞ *This paper will be duly noticed in its turn, while reviewing the volume in which it was originally published.*

### INTELLIGENCE, CORRESPONDENCE, &c.

### LIST

OF

### SUCCESSFUL CANDIDATES

FOR

### THE HOSPITAL REPORT PRIZE.

Palmam qui meruit ferat.

| NO. | NAME. | HOSPITAL. | DATE. |
|---|---|---|---|
| 1 | Thos. H. Smith, (Pupil.) | St. Thomas's. | April, 1827. |
| 2 | Mr. George Bury, (Pupil.) | Winchester Co. | July, 1827. |

Prize—*a complete Set of the* Medico-Chirurgical Review—*perpetual Registry of the successful Candidate's Name in the Journal.*

*N.B. A report came too late. The manuscript must always be forwarded at least one month before publication day.—Two prizes are offered for the succeeding quarter.*

### ADDITIONAL SUBSCRIBERS.

Ashton, Mr. Thomas, Manchester.
Baillie, Mr. Surgeon, Tring, Herts.
Baume, Dr. Berlin.
Beaufoy, Mr. Surgeon, &c.
Bower, Dr. R. Royal Navy, Cape of Good Hope.
Birch, Mr. George, Licentiate of the Apothecaries' Company, Wales.
Carville, Mr. J. W. Surgeon, Knockholt, Kent.
Cooke, Mr. Charles Turner, Member of the Royal College of Surgeons of London, Cheltenham.
Cooke, Mr. John F. Surgeon, Gloucester.
Coull, Dr. Elgin, N.B.
Edwards, Thos. Esq. Surg. Clapham.
Howitt, Mr. E. D. Surg. &c. 3, Apollo Buildings, East-street, Walworth.
Hunter, Samuel, Esq. Glasgow.
Huggins, Daniel, M.D. St. Vincent.
Kennedy, R. H. M.D. Baroda, Bombay
King, Dr. R.N. to New South Wales.
Mackay, Mr. W. Surgeon, Glasgow.
Manthorpe, Mr. D. L. Surgeon, Thorpe le Token, Essex.
Medical Commissioners of His Majesty's Navy, Victualling Board, Somerset Place.
Millar, Dr. S. New Broad-street.*
Morrison, Dr. St. James's Square, London.
Naval Medical Library, Haslar Hospital.
Paul, Dr. John, Elgin, N.B.
Potter, Mr. Surg. Knutsford, Cheshire.
Robertson, Mr. Surgeon, Manchester.
Thomson, Mr. William, Glasgow, (2 copies.)
Wise, Dr. Richard, Helston, Cornwall.

* In No 11 of this Journal, this Gentleman's name was accidentally wrong spelt.

